# Phytochemical Profiling, Antioxidant Activity, and In Vitro Cytotoxic Potential of Mangrove *Avicennia marina*

**DOI:** 10.3390/ph18091308

**Published:** 2025-08-31

**Authors:** Federico Cerri, Beatrice De Santes, Francesca Spena, Lucia Salvioni, Matilde Forcella, Paola Fusi, Stefania Pagliari, Henrik Stahl, Paolo Galli, Miriam Colombo, Marco Giustra, Luca Campone

**Affiliations:** 1 Department of Earth and Environmental Sciences DISAT, University of Milano-Bicocca, Piazza della Scienza 1, 20126 Milan, Italy; federico.cerri@unimib.it (F.C.); b.desantes@campus.unimib.it (B.D.S.); f.spena2@campus.unimib.it (F.S.); paolo.galli@unimib.it (P.G.); 2 MaRHE Centre (Marine Research and Higher Education Center), Magoodhoo Island, Faafu Atoll 12030, Maldives; 3 Department of Biotechnology and Biosciences, University of Milano-Bicocca, Piazza della Scienza 2, 20126 Milan, Italy; lucia.salvioni@unimib.it (L.S.); matilde.forcella@unimib.it (M.F.); paola.fusi@unimib.it (P.F.); miriam.colombo@unimib.it (M.C.); luca.campone@unimib.it (L.C.); 4 Nanomedicine Center NANOMIB, University of Milano-Bicocca, 20854 Vedano al Lambro, Italy; 5 College of Marine Science and Aquatic Biology, University of Khorfakkan, Sharjah 18119, United Arab Emirates; henrik.stahl@ukf.ac.ae

**Keywords:** mangroves, *Avicennia marina*, natural products, bioactive compounds, phytochemical analysis, UPLC-HRMS, antioxidant activity, cytotoxicity, triterpene saponins

## Abstract

**Background:** *Avicennia marina* (Forsk.) Vierh., a widely distributed mangrove species, is known for its diverse secondary metabolites with potential pharmacological applications. Despite its dominance in the Arabian Gulf, where *A. marina* may have adapted to extreme environmental conditions with a distinct set of bioactive molecules, research in this region remains limited. **Methods:** This study investigates the phytochemical composition, antioxidant activity, and in vitro cytotoxicity of extracts from different plant parts, including roots, leaves, propagules, pericarps, and cotyledons, collected in the United Arab Emirates (UAE). Extracts were analyzed using ultra-pressure liquid chromatography coupled with high-resolution mass spectrometry (UPLC-HRMS). Antioxidant activity was assessed using DPPH and ABTS assays, while cytotoxicity was evaluated against human cancer and normal cell lines. **Results:** Analysis revealed 49 compounds, including iridoid glycosides, hydroxycinnamic acids, phenylethanoid glycosides, flavonoid glycosides, and triterpene saponins, several reported for the first time in *A. marina* and mangroves. The pericarp and root extracts exhibited the highest scavenging activity (DPPH: 187.14 ± 2.87 and 128.25 ± 1.12; ABTS: 217.16 ± 2.67 and 147.21 ± 2.42 μmol TE/g, respectively), correlating with phenylethanoid content. The root extract also displayed the highest cytotoxicity, with IC_50_ values of 58.46, 81.98, and 108.10 μg/mL against MDA-MB-231, SW480, and E705, respectively. In silico analysis identified triterpene saponins as potential contributors. **Conclusions:** These findings highlight the root extract of *A. marina* as a promising source of bioactive compounds with potential antioxidant and anticancer applications, supporting further exploration for novel therapeutic candidates.

## 1. Introduction

*Avicennia marina* (Forsk.) Vierh, commonly known as the grey mangrove, is a true mangrove species [1] belonging to the Acanthaceae family. It is widely distributed across tropical and subtropical regions, including Africa; South, Southeast, and Southwest Asia; the Malay Archipelago; the North Island of New Zealand; Australia; the Southwest Pacific; and the Maldives [1,2,3].

The chemical profile of *A. marina* has been extensively studied, leading to the identification of diverse classes of bioactive molecules, including flavonoids, iridoid glycosides, terpenoids, alkaloids, and steroids, as well as a wide range of other metabolites [3,4,5,6,7,8].

Traditionally, *A. marina* has been utilized in folk medicine across different countries to treat several ailments and diseases [9,10]. Furthermore, its extracts and isolated compounds have demonstrated a wide range of biological activities, including antiviral, antimicrobial, anthelmintic and antimalarial, analgesic, antioxidant, antifouling, anticancer, antidiabetic, and anti-inflammatory [3,11]. In particular, extracts of this species have demonstrated promising in vitro anticancer activity [3]. However, research in this field remains relatively limited, especially in the context of the Arabian Gulf, where *A. marina* is the dominant mangrove species along the coasts of the United Arab Emirates (UAE), Saudi Arabia, Bahrain, Qatar, and Iran [12]. Ethnobotanical records for *A. marina* in the Arabian Gulf are scarce but include traditional uses in Iran for treatments of ulcers, rheumatism, and burns [9], and in the UAE for its use as an aphrodisiac, antifertility agent, and treatment for scabies and toothache [13]. *A. marina* remains largely unexplored here in terms of bioprospecting, with most studies focusing instead on its distribution, ecological significance, ecosystem services, and management and conservation [14,15]. Phytochemical investigations have only examined populations from China, India, Pakistan, Egypt, other Indo-Pacific locations, and the Red Sea coasts of Saudi Arabia [10,11,16,17,18].

Previous phytochemical studies on *A. marina* have generally examined only a few plant parts, typically aerial parts and primarily leaves, lacking a comprehensive analysis of plant-part-specific secondary metabolites. Biological activity also presents limitations, often focusing on few plant parts and, in the case of cytotoxicity, testing only a small number of cancer cell lines [9,10,11]. A major gap in existing research of *A. marina* is the absence of integrated studies combining detailed phytochemical profiling with antioxidant and cytotoxic assays across multiple parts of the plant, which is essential for linking bioactivities to tissue-specific metabolites. Such combined strategies are well established in plant research [19,20] and have been applied to mangroves [21] as they facilitate the prioritization of promising extracts and compounds, thereby enhancing efficiency in the early discovery of bioactive molecules. Moreover, the majority of chemical studies have relied on GC-MS, which biases detection towards volatile constituents [3,11,16]. Although GC-MS can also characterize phenolic compounds, abundant in mangroves [22], it requires a derivatization step that is both complex and time-consuming [23]. In contrast, ultra-high performance liquid chromatography coupled with high-resolution mass spectrometry (UPLC-HRMS) has emerged as a powerful platform for untargeted metabolomic profiling of complex plant matrices, offering superior sensitivity, selectivity, and mass accuracy, and enabling comprehensive detection and identification of secondary metabolites, especially phenolics [24,25,26,27].

In addition to these methodological gaps, the limited geographical scope of previous studies represents a crucial limitation in fully understanding the phytochemical diversity and bioactivity of *A. marina*. The Arabian Gulf is characterized by extreme environmental conditions, including elevated seawater temperatures, hypersalinity, and high turbidity, driven by its arid climate and shallow basin [28], and summer air temperatures can reach 50 °C [29], further stressing local ecosystems. Since the chemical composition of plants is influenced by geographical, environmental, and climate factors [30,31,32], plants exposed to such stresses often respond by increasing the accumulation of secondary metabolites, such as flavonoids, iridoid glycosides, and phenylethanoid glycosides, which enhance their tolerance to adverse conditions and also possess bioactivities of pharmacological interest, including antioxidant and anticancer [33,34,35,36,37,38,39].

It is plausible that *A. marina* in the Arabian Gulf may have adapted to extreme conditions through metabolic changes and the induction of antioxidant defense systems [40], potentially resulting in a distinct set of secondary metabolites with unique biological activities. Consequently, the objective of this study is to conduct a comprehensive investigation of *A. marina* grown in the UAE by characterizing the secondary metabolite composition of multiple parts of the plant, including roots, leaves, propagules (pericarps and internal tissues), and cotyledons, and evaluating their potential health benefits through antioxidant and cytotoxic activity assays in vitro.

Unlike previous studies, this work employs UPLC-HRMS to present a detailed phytochemical profile of each tissue type, allowing for the identification of specific compounds of *A. marina* and their localization within the plant. Furthermore, while the antioxidant and anticancer potentials of *A. marina* extracts have been previously reported [3,11], this study provides a comprehensive evaluation of antioxidant and cytotoxic activities of all plant parts, along with expanded cytotoxic screening in multiple cancer cell lines. In addition, in silico predictions of biological activities were applied to compounds identified in the extracts. This multi-level approach addresses existing regional and methodological gaps and lays the groundwork for the discovery of bioactive compounds from plants adapted to extreme environmental conditions.

## 2. Results

### 2.1. Characterization of Avicennia marina Extracts

Roots, leaves, cotyledons, pericarps, and propagules of *A. marina* displayed distinct metabolite profiles. The chromatographic profiles of the extracts are provided in Supplementary Material (Figures S1–S5), and the list of tentatively identified compounds is shown in Table 1, along with their corresponding identification level (IL), which reflects the confidence of compound annotation based on MSI guidelines (see Section 4.4). For compounds assigned to IL2, the identification relied on comparisons of MS/MS fragmentation data with published spectra from the literature or spectral databases. The specific references used to support each IL2 assignment are included directly in the table.

Analysis detected a total of 49 compounds across all plant parts. Triterpene saponins are a heterogeneous secondary metabolite consisting of a terpene-based aglycone linked to one or more sugar chains, commonly glucose (−162 Da), glucuronic acid (−176 Da), and pentoses (−146 Da) [72]. For example, compound 49, which has an m/z of 809.4342 and a molecular formula of C_42_H_66_O_15_, displayed characteristic MS/MS fragments at *m*/*z* 647.3797 [M-H-162] and 471.3469 [M-H-162-176], corresponding to sequential losses of sugar moieties. Based on this fragmentation and the molecular formula, it was identified as Azukisaponin III. Using similar fragmentation patterns, compounds 40, 43, 45, and 46 were also assigned as triterpene saponins [66,67,69,70,71].

Phenylethanoid glycosides are often based on β-D-glucosides of 2-phenylethanol, often with α-L-rhamnose (Rha) substitution at C-3′ of the glucose, resembling variants of verbascoside [73]. Simple phenylethanoid glycosides such as acteoside, isoacteoside, and plantamajoside exhibit similar fragmentation patterns in MS/MS experiments. These are characterized by neutral losses of 162, 152, or 146 *m*/*z*, which are associated with the presence of caffeic acid, glucose, rhamnose, and the phenethanol aglycone. Diagnostic fragment ions at *m*/*z* 179, 161, and 135 indicate the presence of caffeoyl, anhydroglucose, and anhydrophenethanol. Additionally, losses of water (−18 Da) or CO_2_ (−44 Da) are frequently observed [74]. Based on this information, the compounds 7, 13, 14, 17, 20, 22, 23, 24, and 39 belonged to the phenylethanoid glycosides group.

Flavonoid glycosides are a group of secondary metabolites that are widely distributed throughout the plant kingdom. Depending on the bond of the sugar portion, they are divided into *O*-glycosides or C-glycosides and can be distinguished by their unique MS/MS fragmentation spectra, which depend on the nature of the sugar fraction. Generally, C-glycosides exhibit neutral losses of 30, 90, and 120 Da for hexose sugars; 74 and 104 Da for deoxyhexose sugars; and 60 Da for pentose sugars. In contrast, O-glycosides exhibit neutral losses of 162 Da (hexose sugars), 176 Da (glucuronic acid), 146 Da (deoxyhexose sugars), and 132 Da (pentose sugars) [75]. Based on this information, compounds 12, 21, 25, 27, 28, 29, 31, and 37 were identified as O-glycosides.

Iridoid glycosides exhibit distinct fragmentation patterns that depend on the structure of the aglycone ring, the presence of functional groups, and the degree of unsaturation. Typically, a neutral loss of 162 Da is observed, corresponding to the breakage of the bond with the glucoside fraction. Subsequently, the formation of fragments due to the loss of water (18 Da) and the carboxyl group (44 Da) is also observed, together with characteristic fragments resulting from aglycone ring cleavage. Peak 3 with *m*/*z* 373.1139 and molecular formula C_16_H_22_O_10_ was identified as geniposidic acid based on its MS/MS spectrum. Fragment *m*/*z* 211.0605 corresponded to the loss of hexose sugar (162 Da), followed by the presence of fragments *m*/*z* 167.0700 and 149.0597, reflecting subsequent losses of H2O (−18 Da) and CO2 (−44 Da), respectively [76]. Furthermore, fragment 123.0440 is characteristic of the genistein ring. Based on the different fragmentation patterns, compounds 5, 6, 18, 34, and 35 were identified as iridoid glycosides.

Tissue-specific profiling revealed clear metabolic differentiation among parts, with the leaves containing the highest number of secondary metabolites (26), followed by the pericarps (23), roots (23), cotyledons (10), and propagules (6). Notably distinct distribution patterns were observed for specific classes of compounds across the different *A. marina* extracts. The results show that triterpene saponins occur almost exclusively in the root extract (five in roots and only one each in cotyledons, pericarps, and propagules; none in leaves). Phenylethanoid glycosides were predominantly found in root and pericarp extracts (seven in each), with only two detected in leaves and none in cotyledons and propagules. Flavonoid glycosides were mainly associated with leaf extract (six in leaves, two in roots, one in pericarps, and absent in cotyledons and propagules). In contrast, iridoid glycosides and hydroxycinnamic acid and derivatives showed a more uniform distribution across all the extracts.

Analysis confirmed several compounds previously reported in *A. marina*, including caffeoylquinic acid, geniposidic acid, marinoid A, C, and D, acteoside, quercetin 3-*O*-hexoside, kaempferol 3-*O*-glucuronide, isorhamnetin-3-*O*-rutinoside, diosmetin 7-glucuronide, and jionoside C [5,7,77,78,79,80]. Additionally, cistanoside F and kaempferol 3-*O*-glucoside were also detected, previously reported in other mangrove species but not in *A. marina* [81,82]. To our knowledge, several compounds such as mussaenosidic acid, (epi)loganic acid, caffeoylglucaric acid, icariside D1, suspensaside, grandifloroside, suspensaside methyl ether, suspensaside A, isorhamnetin glucuronide, isorhamnetin 7-glucoside, medicoside G, esculentoside C, and azukisaponin III have been newly reported in mangrove species.

### 2.2. Antioxidant Activity

#### 2.2.1. DPPH and ABTS Assays

**Figure 1:**
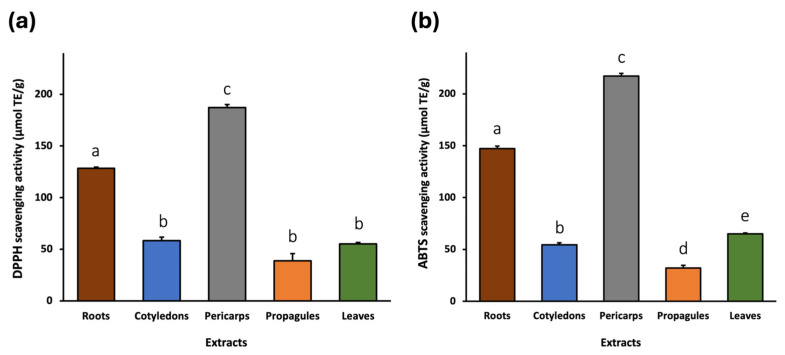
DPPH (**a**) and ABTS (**b**) radical scavenging activity of *A. marina* extracts expressed as μmol Trolox equivalents per gram of sample matrix (μmol TE/g). The bars represent the mean ± standard deviation (SD) from n = 3 independent experiments. Different lowercase letters indicate statistically significant differences between extracts (*p* < 0.05).

The antioxidant potential of *A. marina* extracts was evaluated using two spectrophotometric assays, ABTS and DPPH, which are widely used to assess the free radical scavenging activity of natural compounds. The results are shown in Figure 1.

The DPPH assay showed that the pericarp extract exhibited the highest radical scavenging activity, with a Trolox equivalent antioxidant capacity (TEAC) value of 187.14 ± 2.87 μmol TE/g. This was followed by the extracts of root (128.25 ± 1.12 μmol TE/g), cotyledon (58.23 ± 3.49 μmol TE/g), leaf (55.12 ± 1.52 μmol TE/g), and propagule (38.72 ± 6.96 μmol TE/g).

Similarly, the ABTS assay confirmed that the root and pericarp extracts exhibit high antioxidant activity compared to the other parts of the plant. In fact, the pericarp extracts again displayed the highest TEAC value (217.16 ± 2.67 μmol TE/g), followed by the root (147.21 ± 2.42 μmol TE/g), leaf (64.98 ± 0.84 μmol TE/g), cotyledon (54.46 ± 1.95 μmol TE/g), and propagule (32.23 ± 2.53 μmol TE/g) extracts.

#### 2.2.2. Correlation Between Compound Classes and Antioxidant Activity

The pericarp and root extracts, which exhibited the highest antioxidant activity, are also the ones that contain a high number of phenylethanoid glycosides, compared to the other extracts, which may explain their higher activity. To explore the potential associations between the phytochemical composition of each extract and their antioxidant capacity, a Spearman correlation analysis was performed between the number of compounds in each major chemical class and the measured antioxidant activities (DPPH and ABTS assay) across the five plant-part extracts (n = 5) (Table 2). The analysis revealed a significant positive correlation between the number of phenylethanoid glycosides in the extracts and DPPH activity (ρ = 0.949; *p* = 0.014). ABTS activity showed a similar trend, though the correlation did not reach statistical significance (ρ = 0.791; *p* = 0.111). Antioxidant activity showed no statistically significant correlations with the number of other classes of compounds, including flavonoid glycosides, iridoid glycosides, hydroxycinnamic acids and derivatives, and triterpene saponins (all *p* > 0.05). Given the small sample size and the use of compound counts (not concentrations), these associations should be considered exploratory.

### 2.3. In Vitro Cytotoxic Activity

**Figure 2:**
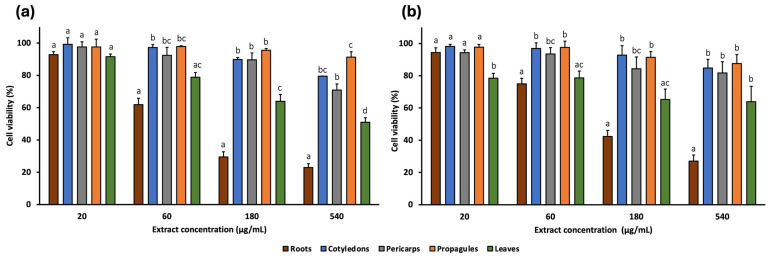
Cell viability (%) of SW480 (**a**) and E705 (**b**) human colorectal cancer cell lines treated with *A. marina* extracts (20–540 μg/mL) for 48 h. Bars represent mean ± standard error of the mean (SEM) from n = 3 independent experiments. Different lowercase letters indicate statistically significant differences between extracts (*p* < 0.05) and were assigned independently for each concentration.

**Figure 3:**
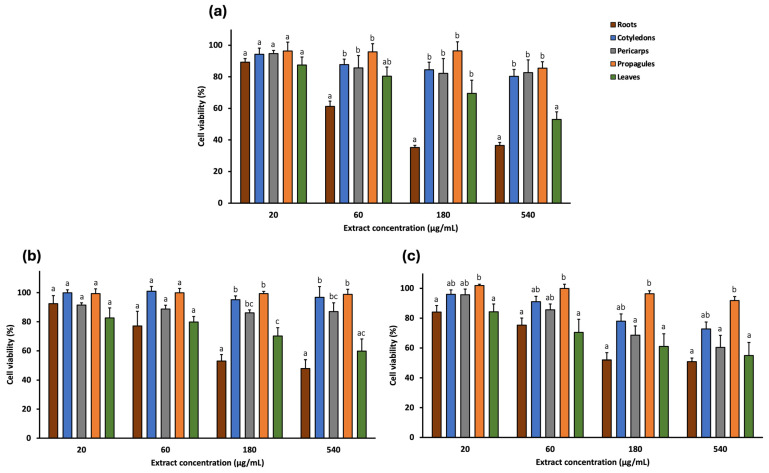
Cell viability (%) of MDA-MB-231 (**a**), U-87 (**b**), and HeLa (**c**) human cancer cell lines treated with *A. marina* extracts (20–540 μg/mL) for 48 h. Bars represent mean ± standard error of the mean (SEM) from n = 3 independent experiments. Different lowercase letters indicate statistically significant differences between extracts (*p* < 0.05) and were assigned independently for each concentration.

**Figure 4:**
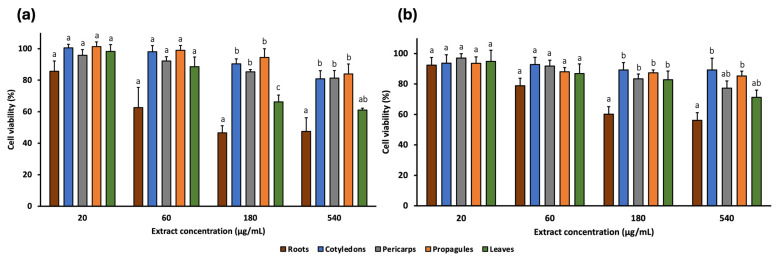
Cell viability (%) of CCD 841 (**a**) and MRC-5 (**b**) healthy human cell lines treated with *A. marina* extracts (20–540 μg/mL) for 48 h. Bars represent mean ± standard error of the mean (SEM) from n = 3 independent experiments. Different lowercase letters indicate statistically significant differences between extracts (*p* < 0.05) and were assigned independently for each concentration.

The cytotoxic effects of *A. marina* extracts (leaf, cotyledon, pericarp, propagule, and root) were evaluated against a panel of human cancer cell lines using the MTT assay. Four concentrations (20, 60, 180, and 540 μg/mL) were tested on two colorectal cancer cell lines (SW480 and E705) (Figure 2) and three additional cancer cell lines: MDA-MB-231 (triple-negative breast cancer), U-87 (glioblastoma), and HeLa (cervical cancer) (Figure 3). Furthermore, two non-cancerous cell lines, MRC-5 (normal human fibroblasts) and CCD 841 (healthy human mucosa), served as controls to assess extract selectivity (Figure 4). The complete data for all concentrations and cell lines are reported in Table S1.

Among the extracts, cotyledon, pericarp, and propagule generally exhibited the lowest cytotoxic activity, reducing cell viability by no more than 70% at the highest concentration (540 μg/mL) across all cell lines. The exception was the pericarp extract, which reduced viability of HeLa cells to 60.30%.

The leaf extract showed low cytotoxicity at lower concentrations. At 60 μg/mL, cell viability remains near 80% for SW480, E705, and MDA-MB-231 and was 70.44% for HeLa. In the non-cancerous cell lines, viability was even higher: 88.59% and 86.86% for CCD 841 and MRC-5, respectively. However, at 540 μg/mL, the extract reduced viability to 50–60% in most cell lines, particularly 50.98% for SW480, 63.91% for E705, 53.00% for MDA-MB-231, 59.77% for U-87, 54.96% for HeLa, and 61.01% for CCD 841, while the least reduction occurred in MRC-5 (71.26%).

Among all extracts, the root extract exhibited the highest cytotoxic activity. At 180 μg/mL, it reduced cell viability to 29.47% (SW480), 42.40% (E705), 35.26% (MDA-MB-231), 53.06% (U87), 52.00% (HeLa), 46.60% (CCD 841), and 60.15% (MRC-5). These reductions were statistically significant compared to all other extracts, except for E705, where differences with the leaf extract were not significant, and for HeLa. At 540 μg/mL, cytotoxicity remained similar across most lines, although viability dropped further in SW480 and E705 (22.93% and 27.03%, respectively).

**Figure 5:**
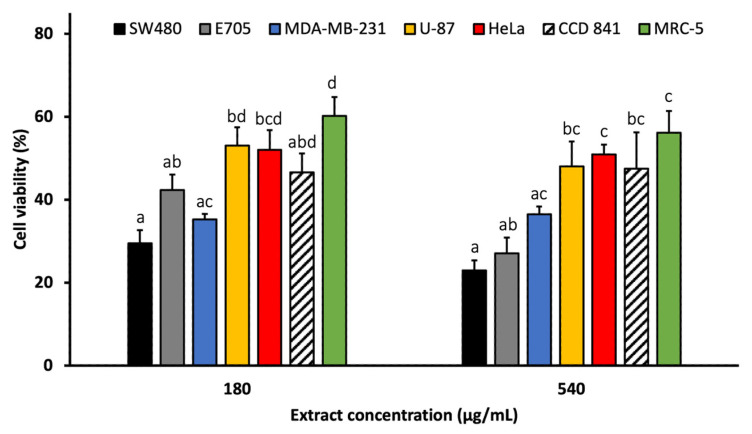
Cell viability (%) of the cancer cell lines treated with *A. marina* root extract (180 and 540 μg/mL) for 48 h. Bars represent mean ± standard error of the mean (SEM) from n = 3 independent experiments. Different lowercase letters indicate statistically significant differences between extracts (*p* < 0.05) and were assigned independently for each concentration.

These findings highlight the notable activity of the root extract, particularly at the highest concentrations, against SW480, E705, and MDA-MB-231 cell lines (Figure 5). Notably, at 180 μg/mL, the cytotoxic activity of the root extract in these cell lines was significantly greater than in non-cancerous MRC-5 cells, while no significant difference was observed compared to the healthy mucosa cell line CCD 841. Additionally, at 540 μg/mL, viability of SW480 cells was significantly lower than that of CCD 841.

Given this pronounced response, the analysis focused on dose–response effects in SW480, E705, and MDA-MB-231. Each cell line was treated with ten increasing concentrations of root extract (2 to 540 μg/mL; Figure S6), and the IC_50_ values were 81.98 μg/mL for SW480, 108.10 μg/mL for E705, and 57.93 μg/mL for MDA-MB-231.

### 2.4. In Silico Analysis

Given the strong cytotoxic activity observed for the *A. marina* root extract in vitro, an in silico analysis was conducted to evaluate the predicted biological activities of the compounds found exclusively or predominantly in this extract. PASS Online predicted potential cytotoxicity-related effects, including antineoplastic activity, apoptosis induction (caspase 3/8 stimulation and apoptosis agonism), TP53 expression enhancement, NF-κB stimulation, cytostatic activity, lipid peroxidase inhibition, and inhibition of ICAM-1 expression [83,84]. The complete list of predicted biological activities for compounds exclusive of the root extract is provided in Table S2 in the Supplementary Materials section.

Among the primary peaks detected in the root extract, the triterpene saponins medicoside G and azukisaponin III were found exclusively in the root extract, while esculentoside C was detected at high levels in the roots and in trace amounts in other extracts. These compounds showed high predicted probabilities (Pa) for antineoplastic activity (0.870, 0.905, and 0.908 for medicoside G, esculentoside C, and azukisaponin III, respectively), caspase 3/8 stimulation (0.994/0.984, 0.989/0.986, and 0.964/0.934), apoptosis agonism (0.901, 0.862, and 0.883), and NF-κB stimulation (0.965, 0.917, and 0.904). They were also predicted to inhibit ICAM-1 expression (0.908, 0.961, and 0.987) and lipid peroxidase activity (0.927, 0.952, and 0.991).

Additional compounds found exclusively in the root extract, including I, suspensaside A, kaempferol 3-O-glucoside, and quercetin 3-O-hexoside, were also predicted to exhibit antineoplastic activity with Pa values of 0.804, 0.863, 0.834, and 0.833, respectively. Kaempferol 3-O-glucoside and quercetin 3-O-hexoside also showed cytostatic activity (Pa: 0.811 and 0.825), enhancement of TP53 expression (Pa: 0.952 and 0.959) and lipid peroxidase inhibition (0.960 and 0.976, resp.).

## 3. Discussion

UHPLC-ESI/HRMS analyses enabled the characterization of *A. marina* extracts, revealing a total of 49 compounds, unevenly distributed across plant parts. The leaf extract contained the highest number of secondary metabolites, followed by pericarps, roots, cotyledons, and propagules. Notably, this study distinguishes between the external tissue of the propagule (here consistently called pericarp) and the internal tissues of the propagule (here consistently called simply propagule) [85], which are often analyzed as a single fruit unit in other studies. The propagule, which consists mainly of the embryo, contained few compounds, likely due to the focus on primary metabolites essential for germination [86]. In contrast, the pericarp, which serves a protective function, was significantly richer in secondary metabolites [87], particularly phenylethanoid glycosides. Similarly, cotyledons had low metabolite diversity and a profile similar to propagules but showed some additional peaks. These may reflect early biosynthesis of stress-related compounds in developing seedlings.

The compounds identified, including phenylethanoid glycosides, flavonoid glycosides, iridoid glycosides, hydroxycinnamic acid and derivatives, and triterpene saponins, are well known for their ecological roles in protecting plants from abiotic stressors such as drought, high salinity, intense sunlight, and elevated temperatures [33,34,35,36,38]. These classes are widely reported in *A. marina* from other regions, and some metabolites identified here have been documented previously, suggesting a shared core phytochemical profile with global populations [10].

However, differences from previous studies were observed. Firstly, a distinctive feature of *A. marina* elsewhere is the presence of naphthalene derivatives [10], which were not detected in our samples. This absence may reflect tissue specificity, as most of these compounds were extracted from branches, or differences in extraction methods [8]. More importantly, several compounds detected in this study have never been reported before in *A. marina*. These include one kaempferol-glycosides and two isorhamnetin glycosides. Notably, monohydroxy B-ring-substituted flavonoid glycosides (e.g., kaempferol-, diosmetin-, and isorhamnetin-glycosides) were more abundant than dihydroxy types, a pattern opposite to that expected under UV stress, where dihydroxy forms typically dominate due to their antioxidant potential [33,35,88]. This shift may reflect Gulf-specific regulation of flavonoid biosynthesis rather than species-specific traits, as it is not evident in previous reports [10]. The study identified novel compounds among the iridoid glycosides and hydroxycinnamic acids, but the most notable findings were within phenylethanoid glycosides and triterpene saponins, the latter all newly reported in *A. marina*. The high abundance of phenylethanoid glycosides in roots is consistent with their reported accumulation under water stress [36], while the accumulation of triterpene saponins is associated with osmotic stress response [38,89]. These trends suggest that UAE-grown *A. marina* may possess a distinctive phytochemical profile shaped by the extreme environmental conditions of the Arabian Gulf [40,90] Nonetheless, inter-regional comparisons are limited due to methodological differences. Future comparative studies using standardized LC-MS protocols would enhance our understanding of mangrove chemical ecology and support bioprospecting efforts.

The identified metabolite classes are associated with various biological activities, including antioxidant, anticancer, antimicrobial, and anti-inflammatory [91,92,93,94,95]. In particular, phenylethanoid glycosides are well-documented antioxidants, showing both DPPH and ABTS radical scavenging activity [91,96,97]. In this study, extracts of the pericarp and root were rich in these compounds and showed the highest antioxidant activity (187.14 ± 2.87 and 217.16 ± 2.67 μmol TE/g for the pericarps; 128.25 ± 1.12 and 147.21 ± 2.42 μmol TE/g for the roots). The strong positive Spearman correlation between the number of phenylethanoid glycosides and DPPH activity (ρ = 0.949; *p* = 0.014) supports the hypothesis that these compounds contribute to radical scavenging in the extracts. Key phenylethanoid glycosides identified in this study, such as cistanoside F, acteoside, and jionoside C, are known for their potent antioxidant activity [98,99,100], while less-studied molecules such as suspensaside and suspensaside A warrant further exploration. Our correlation findings are exploratory and do not prove causation. Definitive attribution will require targeted quantification of candidate phenylethanoid glycosides and subsequent activity testing.

In terms of cytotoxic activity, the cotyledon, pericarp, and propagule extracts showed negligible effects on cancer cell lines, even at the highest concentrations. In contrast, the leaf and root extracts displayed cytotoxicity at higher doses. The leaf extract showed limited cytotoxicity at lower concentrations but reduced viability (50–60%) at 540 μg/mL in several cancer cell lines. This agrees with Momtazi-Borojeni et al. [101], who reported no toxicity at low concentrations but moderate effects at higher doses (250 μg/mL). The root extract had the most promising cytotoxic profile, particularly against SW480, E705, and MDA-MB-231 cancer cell lines. At 540 μg/mL, it reduced cell viability below 40% but showed lower toxicity against normal cell lines (MRC-5 and CCD 841). These findings agree with previous studies showing selective cytotoxicity of root extracts towards cancer cell lines [102]. IC_50_ values for SW480, E705, and MDA-MB-231 were 81.98, 108.10, and 57.93 μg/mL, respectively. Based on the criteria established by the National Cancer Institute (NCI, USA) and the Geran protocol, which classified cytotoxicity as high when IC_50_ values are ≤ 20 μg/mL, moderate between 21 and 200 μg/mL, weak between 201 and 500 μg/mL, and absent above 500 μg/mL [103,104,105], this corresponds to moderate cytotoxic activity. While these IC50 values were not compared with a standard drug, they suggest the presence of active compounds. The values reported here are for crude extracts; further fractionation and isolation of active constituents are expected to yield more potent compounds, for which in vivo and clinical potential could be more realistically assessed through comparison with standard anticancer drugs. These are potentially triterpene saponins, which were only detected in the roots.

Triterpene saponins are gaining attention in cancer research due to their ability to target tumor-related pathways while maintaining low toxicity [106]. Although widespread in medicinal plants [107,108], their occurrence in mangroves is less documented, with only a few studies published on this topic [82,108]. In silico prediction using PASS software indicated a strong cytotoxic potential for several saponins identified in the root extract, including medicoside G, esculentoside C, and azukisaponin III. Additionally, two unidentified saponins suggest the presence of a potentially novel structure that merits further isolation and structural characterization. Other compounds specific to the root extract, such as suspensaside A and kaempferol 3-*O*-glucoside, showed high predicted probabilities for antineoplastic effects.

Given that the root extract exhibited the highest cytotoxicity, further work will focus on bioactivity-guided fractionation of this extract, with particular emphasis on isolating the triterpene saponin-rich fraction. These purified fractions will be tested for cytotoxicity alongside a standard anticancer drug (e.g., doxorubicin) to identify the compounds responsible for the observed activity. Mechanistic studies, including apoptosis assays, cell cycle analysis, and molecular pathway investigations, will be conducted to elucidate the modes of action. Such comprehensive analyses, together with the targeted quantification of candidate constituents, will clarify structure–activity correlations and enhance both therapeutic efficacy and selectivity. Importantly, these efforts, combined with further purification and structural elucidation, could identify promising novel lead compounds suitable for subsequent in vivo evaluation and development as potential anticancer agents.

## 4. Materials and Methods

### 4.1. Chemicals

Ethanol absolute, analytical-grade methanol, 1,1-diphenyl-2-picrylhydrazyl (DPPH^•^), and 2,2-azinobis-(3-ethylbenzothiazoline-6-sulfonate) (ABTS^•+^) reagents were obtained from Sigma-Aldrich (Milan, Italy), while methanol and formic acid of LC-MS grade were sourced from Romil (Cambridge, UK). Ultrapure water (18 MΩ) was prepared by a Milli-Q purification system (Millipore, Bedford, MA, USA).

### 4.2. Plant Material

Samples were collected from different parts of *A. marina*, including leaves, roots, propagules, and cotyledons. The cotyledons were obtained from seedlings at an early growth stage, when the propagules had already opened and developed roots. All samples were harvested in September 2022 from multiple individual plants within the mangrove forest of Ajman Emirate, UAE. Although no herbarium voucher specimen was deposited, the plant material was identified based on morphological characteristics following established taxonomic keys [109]. This identification is supported by the fact that *A. marina* is the only mangrove species forming the evergreen coastal forests of the UAE [14], and its presence in the region has been validated by previous molecular analyses [110,111].

For more precise phytochemical characterization, propagules were separated into the pericarp, representing the external protective tissue, and the internal tissues. Thus, in the manuscript, the term pericarp (and pericarp extract) refers exclusively to the external part, while the generic term propagule (and propagule extract) refers to the internal tissues. Each type of plant material (e.g., all collected leaves, roots, pericarps, propagules, and cotyledons) was pooled by type and immediately freeze-dried after collection. The dried samples were homogenized using a Grindomix GM 200 knife mill (Retsch, Haan, Germany) and then sieved through a test sieve (Retsch AS 200, Haan, Germany) with a mesh size range of 300–600 μm to obtain powders with uniform particle size distribution.

### 4.3. Sample Preparation and Extraction

Root, leaf, cotyledon, pericarp, and propagule samples of *A. marina* underwent exhaustive ultrasound-assisted extraction using a thermostatically controlled ultrasonic bath (Sonorex TK 52; Bandelin electronic, Berlin, Germany). Each sample was extracted under controlled conditions (25 °C, 15 min) with 50% aqueous ethanol (*v*/*v*) at a solid-to-solvent ratio of 1:10 (*w*/*v*), which is commonly applied in metabolite profiling studies [112]; specifically, 1 g of powdered sample was mixed with 10 mL of solvent in a 50-mL polypropylene tube. A 50% aqueous ethanol solution was selected as the extraction solvent due to its effectiveness as a green, low-toxicity system, making it well-suited for bioactivity screening [113,114]. Ethanol–water mixtures offer a balanced polarity and are widely recognized for their ability to efficiently extract a broad range of bioactive compounds, particularly polyphenolic metabolites, which are well known for their antioxidant properties [115,116]. Moreover, this solvent was selected to ensure low toxicity in downstream biological assays, in case traces of solvent remain after evaporation, and because non-polar solvents or higher ethanol concentrations could reduce solubility in aqueous assay media, potentially compromising the suitability of the extracts for biological testing.

To ensure complete extraction, the process was repeated three times with fresh solvent. Following each extraction, the mixtures were centrifuged (13,000× *g*, 10 min), and the supernatants underwent filtration through Whatman No. 1 filter paper. The combined extracts were concentrated under pressure at 40 °C using a rotary evaporator to remove ethanol and subsequently lyophilized (Alpha 1-2 LD freeze dryer, Christ, Germany) to obtain dry residues for further analysis.

The extraction yields of *A. marina* were determined by calculating the ratio of the weight of dried extract obtained to the initial weight of dried plant material powder and expressed as a percentage. The yields were 34.23% for roots, 65.02% for cotyledons, 61.76% for pericarps, 38.55% for propagules, and 33.72% for leaves.

### 4.4. Characterization of Extracts

The chemical characterization of extracts was performed in negative mode using liquid chromatography coupled with electrospray ionization (ESI) and high-resolution mass spectrometry (UPLC-ESI/HRMS). A Waters ACQUITY UPLC system coupled with a Waters Xevo G2-XS QTof Mass Spectrometer (Waters Corp., Milford, MA, USA) was used. The extracts were dissolved in ultrapure water at a concentration of 100 μg/mL, and then 5 μL of each sample was injected into a Biphenyl column (100 mm × 2.1 mm, 2.6 μm; Phenomenex, Torrance, CA, USA). The chromatographic gradient was conducted with solvent A (0.1% formic acid in water) and solvent B (0.1% formic acid in methanol), starting with 95% A for 1 min, followed by a linear gradient to 95% B over 10 min, and 4 min of column washing at 95% B. The flow rate was maintained at 0.4 mL/min. The ESI source was operated under the following conditions: electrospray capillary voltage of 1.5 kV, source temperature of 140 °C, and desolvation temperature of 600 °C. MS spectra were acquired in full range mode, covering a mass range of 100–1000 *m*/*z*. MS/HRMS analysis was performed using data-dependent scan (DDA), selecting the two most intense ions from the HRMS scan for collision-induced dissociation (CID) with the following conditions: a minimum signal threshold of 500,000, isolation width at 2.0, and normalized collision energy of 30%. Metabolite identification followed the Metabolomics Standards Initiative (MSI) guidelines, which define three confidence levels indicated in the “IL” column of Table 1: Level 1 (IL1): compounds were unequivocally identified by comparison with authentic reference standards (retention time, MS/MS spectrum, and exact mass); Level 2 (IL2): tentative identifications were assigned based on matches between experimental MS/MS spectra and literature data or spectral libraries (e.g., GNPS, MassBank); Level 3 (IL3): compounds were classified by spectral similarity to known chemical families and supported by taxonomic evidence.

Novelty verification was performed using general literature databases (e.g., Google Scholar) and the chemical database SciFinder^n^. In SciFinder^n^, each tentatively identified compound was queried by chemical name, and all related references were investigated using keywords such as “*Avicennia marina*”, “*Avicennia*”, and “mangroves”. This process allowed determination of whether a compound had been previously reported in *A. marina*, other species within the genus *Avicennia*, or other mangrove species, or if it represents a first identification in mangroves.

### 4.5. Determination of Antioxidant Activity

The antioxidant capacities (AOCs) of the exhaustive extracts of *A. marina* (leaves, roots, pericarps, propagules, and cotyledons) were evaluated using 1,1-diphenyl-2-picrylhydrazyl (DPPH^•^) and 2,2-azinobis-3-ethylbenzothiazoline-6-sulfonate (ABTS^•+^) assays according to Cannavacciuolo et al. [117]. The extracts were dissolved in ultrapure water and analyzed at a concentration of 0.5 mg/mL, with Trolox (0–500 μM) serving as a standard. The antioxidant activity was expressed as μmol Trolox equivalents per gram of sample matrix (TE/g MTX), representing the μmol of a standard Trolox solution exerting the same antioxidant capacity as 1 mg/mL of the tested extracts.

In the DPPH assay the stock solution of DPPH (5 mM) was prepared by dissolving 3.9 mg of DPPH in 100 mL of methanol and subsequently diluted to 100 μM to obtain the operating solution. This solution was prepared just before use and protected from light due to the photosensitivity of the reagent. The assay was set up in an Eppendorf by mixing 50 μL of sample with 950 μL of operative DPPH, and the mixture was incubated in the dark for 30 min. Subsequently, 200 μL of the solution was transferred to an absorbance reading plate at the 515 nm wavelength.

For the ABTS assay, the stock solution of ABTS (7 mM) was diluted with phosphate-buffered saline (PBS; 5 mM, pH 7.4) to achieve working concentrations. The assay was performed by combining 5 μL of diluted sample (or PBS control) and 500 μL of ABTS radical cation solution (0.1 mM in PBS). The reaction mixtures were protected from light and incubated at 30 °C for 60 min to allow complete radical scavenging. Absorbance measurements were then recorded at 734 nm using a microplate reader.

### 4.6. Cytotoxicity Evaluation

#### 4.6.1. Cell Lines and Culture Conditions

Normal human fibroblasts (MRC-5), human glioblastoma (U-87), human triple-negative breast cancer (MDA-MB-231), human colorectal cancer (SW480), and human healthy mucosa (CCD841) cell lines were purchased from the American Type Culture Collection (Manassas, VA, USA). The human cervical cancer cell line (HeLa) was acquired from System Biosciences, and the human colon cancer cell line (E705) was provided by the Fondazione IRCCS Istituto Nazionale dei Tumori (Milan, Italy). The E705 cell line represents epithelial tissue cells of colorectal adenocarcinoma derived from a patient at the National Cancer Institute in Milan.

MRC-5, U-87, and HeLa cells were cultured in Dulbecco’s Modified Eagle Medium (DMEM) high glucose medium supplemented with 10% heat-inactivated fetal bovine serum (FBS), 1% penicillin/streptomycin (P/S), and 2 mM L-glutamine. MDA-MB-231 cells were maintained in Minimum Essential Medium (MEM) with Earl’s Salts supplemented with 10% heat-inactivated FBS, 1% P/S, 2 mM L-glutamine, and 0.1 mM MEM Non-Essential Amino Acids (MEM NEAA). E705 and SW480 cell lines were cultured in RPMI 1640 medium supplemented with 10% heat-inactivated FBS, 100 U/mL penicillin, 100 μg/mL streptomycin, and 2 mM L-glutamine. The CCD 841 cell line was grown in EMEM medium supplemented with 10% heat-inactivated FBS, 1% P/S, 2 mM L-glutamine, and 0.1 mM non-essential amino acids. All cell lines were incubated at 37 °C in a humidified atmosphere containing 5% CO_2_ and 95% air. Cell culture media and reagents were purchased from EuroClone (Pero, Italy).

#### 4.6.2. Viability Assay

The cytotoxicity of *A. marina* extracts was evaluated using the MTT assay (CellTiter96^®^Non-Radioactive Cell Proliferation Assay, Promega, Madison, WI, USA) following the manufacturer’s protocol. The extract powders were solubilized in Milli-Q water, and four extract concentrations (20, 60, 180, and 540 μg/mL) were tested on all cell lines. Additionally, for the root extract, an extended dose-response analysis using ten concentrations (2, 10, 20, 40, 60, 100, 140, 180, 360, and 540 μg/mL) was performed on selected cell lines to enable IC_50_ determination. IC_50_ values were calculated only for extract–cell line combinations tested with this ten-point dilution series.

No positive control drugs were included in this preliminary screening, as the primary objective was to assess and compare the relative cytotoxicity of different *A. marina* extracts. Comparative analysis with standard anticancer agents will be incorporated in subsequent studies on purified fractions or isolated compounds.

Briefly, HeLa, MRC-5, U-87, and MDA-MB-231 cells were seeded into 96-well plates (from Euroclone, Pero, Italy) at a density of 5 × 10^3^ cells/well in 100 μL of growth medium, while E-705 and SW-480 cells were seeded at a density of 8 × 10^3^ cells/well. After 24 h of incubation at 37 °C in 5% CO_2_, the medium was replaced, and cells were treated with various concentrations of *A. marina* extracts. Following 48 h of treatment, the medium was replaced, and 15 μL of MTT solution was added to each well. After 3 h of incubation at 37 °C, formazan crystals were solubilized using 100 μL of stop solution and incubated under stirring for 1 h. Reduced MTT was quantified using a UV–vis plate reader (EnSight Multimode Microplate Reader, PerkinElmer, Waltham, MA, USA) at 570 nm with a reference wavelength of 630 nm.

Cell viability was expressed as a percentage relative to untreated cells (negative control), and medium with MilliQ water at equivalent concentrations (10% *v*/*v*) was used as a blank. Dose-response curves and the IC_50_ values, representing the extract concentration required to inhibit 50% of cell viability relative to untreated control cells, were generated using GraphPad Prism v10.5.0 software.

### 4.7. In Silico Prediction for Anticancer Activity

To identify potential bioactive compounds responsible for the cytotoxic effect, an in silico prediction of biological activity was performed using PASS Online software (https://www.way2drug.com/PASSOnline/index.php; accessed on 15 July 2025), a predictive tool from Way2Drug Services. The reliability of PASS for predicting in vitro cytotoxic activity has been demonstrated in previous studies [118,119], including those focusing on triterpene saponins [120].

The canonical Simplified Molecular Input Line Entry System (SMILES) of each compound was gathered from SciFinder_n_ and was used to run the software. The program independently calculates the estimated predictive biological activities based on structure–activity relationships, providing Pa (probability of activity) and Pi (probability of inactivity) values for each activity. Only activities with Pa > 0.7 were considered, as this threshold indicates a high likelihood that the substance will exhibit the predicted activity in experimental settings, although the probability of the compound being an analogue of a known pharmaceutical agent remains high [121]. Notably, when Pa > 0.9, as frequently observed in our study, the likelihood of false-positive predictions is insignificant [118].

The predicted anticancer-related activities included antineoplastic activity, apoptosis-related effects (apoptosis agonist, caspase 3/8 stimulation), TP53 expression enhancement, NF-kB modulation, cytostatic activity, lipid peroxidase inhibition, and ICAM-1 expression inhibition. These results were analyzed in relation to the cytotoxicity data of the MTT assay to establish potential correlations between the phytochemical composition and the observed cytotoxicity against cancer cells.

### 4.8. Statistical Analysis

Statistical analyses were conducted on data generated from three replicates. Cytotoxic activity results are presented as mean ± standard error of the mean (SEM). Antioxidant activity results are reported as mean ± standard deviation (SD). Before statistical analysis, the assumption of normality was assessed using the Shapiro–Wilk test. The homogeneity of the variances was evaluated using Levene’s test. When normality and homogeneity assumptions were met, a one-way analysis of variance (ANOVA) was performed, followed by Tukey’s honest significant difference (HSD) post hoc test to assess pairwise differences between the means of the group. In cases where the assumption of homogeneity of the variances was not met, Welch’s ANOVA was applied, followed by the Games–Howell post hoc test. Statistical significance was considered when *p* < 0.05.

The correlation between phytochemical composition and antioxidant activity was assessed using Spearman’s rank correlation coefficient (two-tailed). For each plant-part extract (n = 5), the number of tentatively identified compounds in each major chemical class (phenylethanoid glycosides, flavonoid glycosides, iridoid glycosides, hydroxycinnamic acids and derivatives, and triterpene saponins) was correlated with antioxidant activity (DPPH and ABTS, mean values). Statistical significance was set at *p* < 0.05.

All analyses were conducted with IBM SPSS Statistics v29.0.2.0.

## 5. Conclusions

The findings of this study highlight *A. marina* as a valuable source of bioactive compounds with promising therapeutic applications. The pericarp and root extracts exhibited the highest antioxidant activity, possibly due to the presence of phenylethanoid glycosides, which are known for their antioxidant activities. Among all extracts tested, the root extract displayed the strongest cytotoxicity, in particular against the triple-negative breast cancer cell line MDA-MB-231 and two colorectal cancer cell lines, SW480 and E705, with IC_50_ values of 58.46, 81.98, and 108.10 μg/mL, respectively. In silico predictions identified triterpene saponins, including medicoside G, esculentoside C, and azukisaponin III, as likely contributors to these effects.

The detection of triterpene saponins not previously reported from mangroves, together with several phenylethanoid glycosides and other compounds not earlier described in *A. marina*, is noteworthy. Plants adapted to extreme environments can accumulate distinctive secondary metabolites, and our plant-part-specific UPLC-HRMS analysis of UAE-grown *A. marina* provides region-specific evidence that complements existing phytochemical surveys. Moreover, combining this untargeted phytochemical investigation with biological activity screening and in silico analysis/statistical correlation offers a practicable approach to rapidly link observed activities to plant-part-specific compounds.

To advance these observations towards pharmacological relevance, future work should focus on bioactivity-guided fractionation of the extracts, structural elucidation, and targeted quantification of key compounds, along with mechanistic in vitro assays. These efforts will help clarify structure–activity correlations and potentially lead to the identification of a novel therapeutic candidate from this stress-adapted mangrove species.

## Figures and Tables

**Table 1:** UHPLC-ESI/HRMS data of compounds detected in *Avicennia marina* extracts. The main fragment ion for each compound is indicated in bold.

No.	RT (min)	[M − H]^−^	Formula	Δ ppm	MS/MS	Name	Class	Part	IL	Ref.
1	0.58	701.1893 [M + Cl]^−^	C_24_H_42_O_21_	−2.1677	665.2134, 485.1499, 443.1393, **383.1182**, 341.1075, 179.0549	Stachyose	Tetrasaccharides	Cotyledons/pericarps/propagules/roots	IL2	[41]
2	0.99	191.0188	C_6_H_8_O_7_	6.3850	111.0073	Citric acid	Tricarboxylic acids	Cotyledons/pericarps	IL2	[42]
3	3.87	373.1139	C_16_H_22_O_10_	0.3221	211.0605, 167.0700, 149.0597, **123.0440**, 105.0333	Geniposidic acid	Iridoid glycosides	Leaves/cotyledons/pericarps/propagules/roots	IL2	[43]
4	4.03	353.0875	C_16_H_18_O_9_	0.8633	**191.0551**, 179.0339, 161.0233, 135.0439	Caffeoylquinic acid isomer	Hydroxycinnamic acids and derivatives	Roots	IL2	[44]
5	4.05	375.1291	C_16_H_24_O_10_	1.5167	213.0756, 169.0857, **151.0753**, 133.0644, 125.0595, 107.0490	Mussaenosidic acid	Iridoid glycosides	Leaves/cotyledons/pericarps/propagules	IL2	[45]
6	4.33	375.1285	C_16_H_24_O_10_	3.1119	213.0747, 169.0854, **151.0748**, 133.0644, 125.0591, 113.0230, 107.0484	(Epi)loganic acid	Iridoid glycosides	Leaves	IL2	[45]
7	4.53	487.1451	C_21_H_28_O_13_	1.2588	**179.0334**, 161.0228, 135.0435	Cistanoside F	Phenylethanoid glycosides	Pericarps	IL2	[46]
8	4.80	327.0715	C_14_H_16_O_9_	1.9983	179.0335, 165.0389, 147.0283, **135.0434**, 105.0178	Unidentified	-	Leaves		
9	5.09	353.0871	C_16_H_18_O_9_	−3.0904	**191.0550**, 179.0337, 173.0442, 161.0232, 135.0439,	Caffeoylquinic acid isomer	Hydroxycinnamic acids and derivatives	Leaves/cotyledons/pericarps/propagules/roots	IL2	[44]
10	5.47	371.0982	C_15_H_16_O_11_	−8.7858	**209.0635**, 179.0337, 161.0228, 135.0435, 129.0178	Caffeoyl hexaric acid	Hydroxycinnamic acids and derivatives	Leaves	IL2	[47]
11	5.60	443.0655	C_18_H_20_O_11_S	−0.3243	275.0218, **167.0338**, **152.0105**, 123.0440, 108.0204	Unidentified	-	Roots		
12	5.65	415.1603	C_19_H_28_O_10_	1.6114	235.0963, **191.1062**, 173.0958, 149.0953, 137.0590, 101.0226	Icariside D1	Flavonoid glycosides	Leaves	IL2	[48]
13	5.93	639.1964	C_29_H_36_O_16_	−5.2193	621.1807, 529.1554, 459.1488, 251.0549, 179.0337, **161.0232**, 151.0387	Suspensaside isomer	Phenylethanoid glycosides	Pericarps/roots	IL2	[49,50]
14	5.95	639.1964	C_29_H_36_O_16_	−5.2193	621.1807, 529.1554, 459.1488, 251.0549, 179.0337, **161.0232**, 151.0387	Suspensaside isomer	Phenylethanoid glycosides	Pericarps/roots	IL2	[49,50]
15	6.14	537.1628	C_25_H_30_O_13_	−2.6672	493.1708, 375.1275, 323.0758, 213.0752, 179.0334, 169.0854, **161.0230**, 151.0750, 135.0435, 125.0593, 107.0486	Grandifloroside	Hydroxycinnamic acid and derivatives	Leaves	IL2	[51]
16	6.33	619.1644	C_29_H_32_O_15_	3.9407	383.0758, **311.0549**, 267.0646	Unidentified	-	Pericarps/roots		
17	6.41	639.1929	C_29_H_36_O_16_	0.2477	**621.1817**, 529.1554, 459.1493, 251.0549 **179.0338**, 161.0236, 151.0385	Suspensaside isomer	Phenylethanoid glycosides	Roots	IL2	[49,50]
18	6.65	521.1658	C_25_H_30_O_12_	1.2446	357.1176, 169.0854, 163.0385, 151.0749, 145.0280, 125.0591, 119.0486, 117.0329, 107.0486	Marinoid C	Iridoid glycosides	Leaves/cotyledons/pericarps	IL3	[7]
19	6.65	653.2091	C_29_H_34_O_17_	9.55333	621.1822, 459.1499, 179.0338, **161.0234**, 151.0388, 135.0437	Suspensaside methyl ether	Phenylethanoid glycosides	Roots	IL2	[49,50]
20	6.79	623.1981	C_29_H_36_O_15_	0.0705	461.1657, **161.0233**, 113.0283	Verbascoside (acteoside) isomer	Phenylethanoid glycosides	Leaves/pericarps/roots	IL2	[52]
21	6.9	463.0874	C_21_H_20_O_12_	1.7229	301.0324, **300.0264,** 271.0235, 255.0285	Quercetin 3-*O*-hexoside	Flavonoid glycosides	Roots	IL2	[53,54,55]
22	7.02	667.2239	C_31_H_40_O_16_	0.6864	621.1824, 459.1499, **179.0338**, **161.0235**, 151.0386, 135.0436	β-ethyl-OH-verbascoside	Phenylethanoid glycosides	Pericarps	IL2	[56,57]
23	7.11	623.2001	C_29_H_36_O_15_	−3.1336	461.1661, **161.0235**,	Verbascoside (acteoside) isomer	Phenylethanoid glycosides	Leaves/pericarps/roots	IL2	[52]
24	7.21	621.1838	C_29_H_34_O_15_	−2.0992	461.1652, 179.0337, **161.0233**, 151.0387	Suspensaside A	Phenylethanoid glycosides	Roots	IL2	[49,50]
25	7.21	461.0718	C_21_H_18_O_12_	1.6222	**285.0391**	Kaempferol-3-*O*-glucuronide	Flavonoid glycosides	Leaves	IL2	[58]
26	7.21	681.2063	C_31_H_38_O_17_	−3.9236	519.1708, 490.1321, 181.0129, 179.0334, **161.0230**	Unidentified	-	Pericarps		
27	7.3	447.0926	C_21_H_20_O_11_	1.5287	327.0494, 285.0648, **284.0315**, 255.0288, 227.0338, 151.0013	Kaempferol 3-*O*-glucoside	Flavonoid glycosides	Roots	IL2	[55,59,60,61]
28	7.40	623.1658	C_28_H_32_O_16_	−6.4750	**315.0494**, 314.0421, 300.0258, 299.0187, 271.0234	Isorhamnetin-3-*O*-rutinoside	Flavonoid glycosides	Leaves/pericarps	IL2	[62]
29	7.40	491.0828	C_22_H_20_O_13_	0.6385	**315.0499**, 300.0264	Isorhamnetin glucuronide	Flavonoid glycosides	Leaves	IL2	[63]
30	7.51	535.1477	C_25_H_28_O_13_	−3.7032	329.1021, **179.0338**, 161.0232, 149.0595, 135.0438	Unidentified	-	Leaves/Pericarps		
31	7.51	477.1036	C_22_H_22_O_12_	−5.1250	315.0467, **314.0420**, 285.0392, 271.0236, 257.0441, 243.0286,	Isorhamnetin 7-glucoside	Flavonoid glycosides	Leaves	IL2	[63]
32	7.61	471.1874	C_22_H_32_O_11_	−0.4545	287.1273, **263.1278**, 219.1379, 201.1273, 186.1036, 147.1166	Unidentified	-	Pericarps		
33	7.86	519.1143	C_24_H_24_O_13_	0.2199	315.0472, **314.0423**, 299.0186, 285.0383, 271.0236, 257.0443, 243.0286	Unidentified	-	Leaves		
34	7.90	553.1556	C_25_H_30_O_14_	1.2256	329.1021, **197.0445**, 182.0206, 153.0454, 149.0596, 131.0489,	Marinoid D	Iridoid glycosides	Cotyledons/pericarps/propagules/roots	IL3	[7]
35	7.95	505.1757	C_25_H_29_O_11_	0.2000	357.1184, 213.0757, 195.0650, 169.0857, 151.0753, **147.0439**, 125.0596, 113.0230, 107.0487, 103.0539	Marinoid A	Iridoid glycosides	Leaves	IL3	[7]
36	8.03	519.1505	C_25_H_28_O_12_	0.5766	313.1072, 295.0961, **163.0388**, 149.0596, 145.0282, 131.0490, 119.0487	Unidentified	-	Leaves/cotyledons/pericarps/roots		
37	8.03	475.0887	C_22_H_20_O_12_	−1.0510	300.0589, **299.0554**, 285.0358, **284.0318.**	Diosmetin 7-glucuronide	Flavonoid glycosides	Leaves	IL2	[64]
38	8.24	549.1616	C_26_H_30_O_13_	−0.4279	343.1176, 325.1064, **193.0495**, 175.0387, 149.0595, 134.0360, 131.0489	Unidentified	-	Leaves/cotyledons/pericarps/roots		
39	8.36	591.2119	C_29_H_36_O_13_	−6.0539	179.0333, **161.0234**, 133.0282, 113.0228	Jionoside C	Phenylethanoid glycosides	Pericarps	IL2	[65]
40	8.62	825.4276	C_44_H_66_O_16_	0.2536	**663.3744**, 601.3735	Unknown triterpene saponin	Triterpene saponins	Roots	IL3	[66,67]
41	8.70	539.2152	C_26_H_36_O_12_	−3.3318	193.0485, 183.1010, 175.0382, 149.0591, 131.0485, 121.0642	Unidentified	-	Leaves		
42	8.80	541.2285	C_26_H_38_O_12_	1.0147	193.0485, 185.1166, 175.0382, 149.0591, 131.0485, 121.0642	Unidentified	-	Leaves		
43	8.88	825.4285	C_42_H_66_O_16_	−0.8354	**663.3744**, 601.3735, 487.3421	Unknown triterpene saponin	Triterpene saponins	Roots	IL3	[66,67]
44	8.97	299.0546	C_16_H_12_O_6_	5.0379	285.0345, **284.0313**, 256.0363, 227.0334	Trihydroxy-methoxyflavone	Flavones	Leaves	IL2	[68]
45	8.99	825.4273	C_42_H_66_O_16_	0.6166	**663.3744**, 601.3735,	Medicoside G (medicagenic acid 3,28-di-glucoside)	Triterpene saponins	Roots	IL2	[66,67]
46	9.08	809.4316	C_42_H_66_O_15_	1.5979	689.3884, **647.3788**, 629.3680, 585.3786	Esculentoside C (phycolaccoside D)	Triterpene saponins	Cotyledons/pericarps/propagules/roots	IL2	[69,70]
47	9.30	505.1711	C_25_H_30_O_11_	0.8600	281.1170, 195.0649, 151.0750, **147.0438**, 133.0645, 107.0486	Unidentified	-	Leaves		
48	9.38	503.1572	C_25_H_28_O_11_	−2.6077	279.1010, 253,0854, 209.0954, 195.0647, **147.0437**, 131.0486, 103.0536	Unidentified	-	Leaves/pericarps		
49	9.54	809.4342	C_42_H_66_O_15_	−1.6102	**647.3797**, 471.3469	Azukisaponin III	Triterpene saponins	Roots	IL2	[71]

**Table 2:** Spearman correlation coefficients (ρ) between the number of compounds per chemical class and the antioxidant activity (DPPH and ABTS assays). Statistically significant correlations are indicated in bold (*p* value < 0.05).

Compound Class	DPPH		ABTS	
	ρ-Value	*p*-Value	*p*-Value	*p*-Value
Iridoid glycosides	−0.103	0.870	0.510	0.935
Hydroxycinnamic acid and derivatives	−0.112	0.858	0.224	0.718
Phenylethanoid glycosides	0.791	0.111	0.949	**0.014**
Flavonoid glycosides	0.205	0.741	0.574	0.322
Triterpene saponins	0.447	0.450	0.224	0.718

## Data Availability

Data presented in this study is contained within the article and Supplementary Materials. Further inquiries can be directed to the corresponding author.

## Supplementary Materials

The following supporting information can be downloaded at: https://www.mdpi.com/article/10.3390/ph18091308/s1, Figures S1–S5: Representative chromatograms of *A. marina* extracts; Table S1: Cytotoxicity of *Avicennia marina* extracts on the tested cell lines at four concentrations (20–540 μg/mL); Figure S6: Cell viability of SW480 (a), E705 (b), and MDA-MB-231 (c) human cancer cell lines treated with root extract (2–540 μg/mL) for 48 h; Table S2: Probable cytotoxicity-related biological activities of 6 compounds tentatively identified in the root extract of *A. marina* by PASS (Prediction of Activity Spectra for Substances).
