# Supplementary material for: Phytochemical Profiling, Antioxidant Activity, and In Vitro Cytotoxic Potential of Mangrove Avicennia marina
Source: Pharmaceuticals (Basel). 2025 Aug 31;18(9):1308. doi: 10.3390/ph18091308 (PMC12472679; doi:10.3390/ph18091308)
Supplement: Supplementary file 1 [file pharmaceuticals-18-01308-s001.zip › pharmaceuticals-3800846-supplementary.pdf]

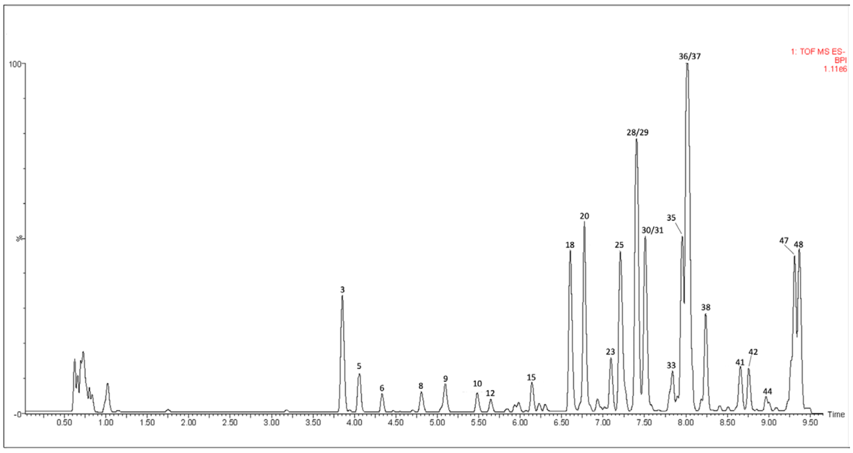

Figure S1. Representative chromatogram of the leaf extract of *A.marina.*

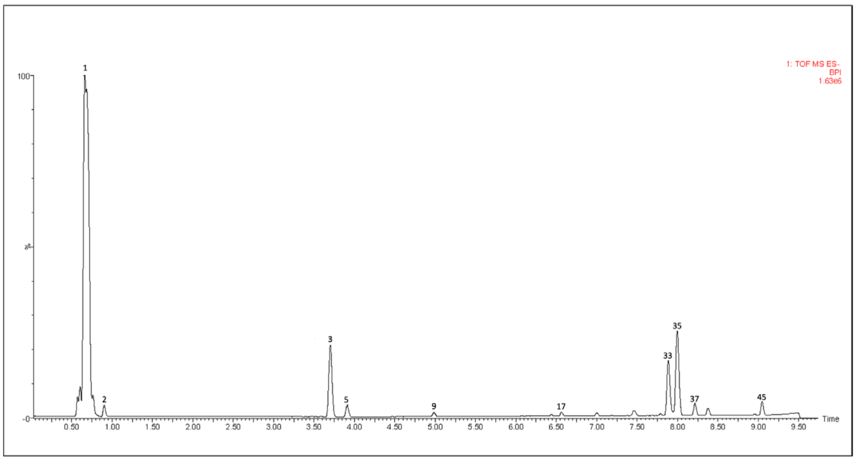

Figure S2. Representative chromatogram of the cotyledon extract of *A.marina.*

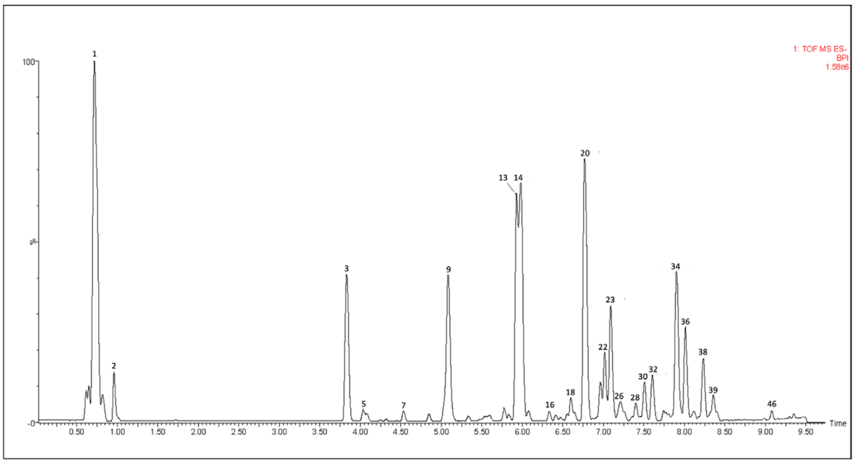

Figure S3. Representative chromatogram of the pericarp extract of *A.marina.*

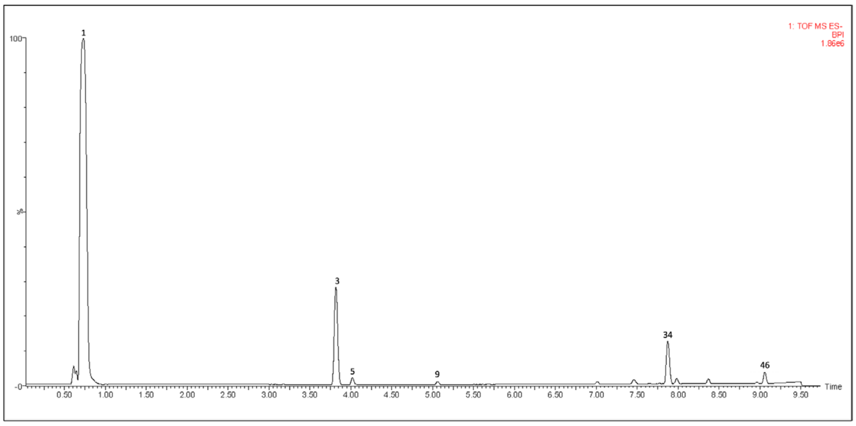

Figure S4. Representative chromatogram of the propagule extract of *A.marina.*

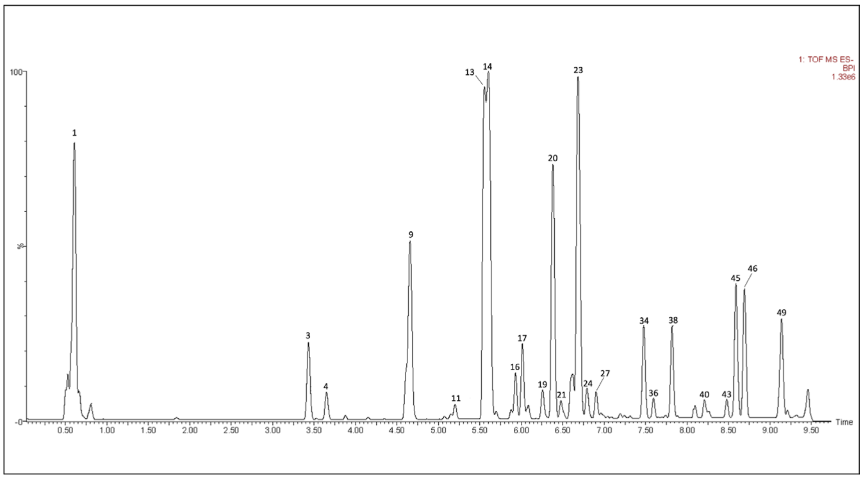

Figure S5. Representative chromatogram of the root extracts of *A.marina.*

Table S1. Cytotoxicity of *Avicennia marina* extracts on the tested cell lines at four concentrations (20–540µg/mL). Cell viability is expressed as a percentage relative to untreated cells (negative control). Standard error of the mean (SEM) is also reported.

Cell line	Extract conc. (µg/mL)	Roots		Cotyledons		Pericarps		Propagules		Leaves	
		Cell viability (%)	SEM	Cell viability (%)	SEM	Cell viability (%)	SEM	Cell viability (%)	SEM	Cell viability (%)	SEM
**SW480**	**20**	92.84	1.83	99.21	4.09	97.64	3.19	97.62	4.84	91.61	1.55
	**60**	61.92	4.00	97.25	1.85	92.42	4.93	97.89	0.49	78.80	3.02
	**180**	29.47	3.20	89.80	1.27	89.63	4.27	95.61	1.07	63.95	4.28
	**540**	22.93	2.42	79.51	0.15	70.95	3.80	91.30	3.32	50.98	2.82
**E705**	**20**	94.40	2.85	98.20	1.24	94.33	1.71	97.74	1.64	78.50	2.93
	**60**	74.94	3.42	96.94	3.50	93.48	4.00	97.59	3.86	78.67	4.26
	**180**	42.30	3.77	92.77	5.92	84.34	7.33	91.45	3.48	65.22	6.57
	**540**	27.03	3.81	84.76	5.44	81.71	6.96	87.57	5.58	63.91	9.56
**MDA-MB-231**	**20**	89.26	2.25	94.24	3.95	94.68	2.07	96.37	5.59	87.46	5.05
	**60**	61.27	3.37	87.76	3.46	85.62	7.82	95.83	5.16	80.37	5.79
	**180**	35.26	1.30	84.44	4.81	82.19	9.32	96.41	5.75	69.46	8.36
	**540**	36.47	1.90	80.29	4.39	82.62	8.10	85.50	4.03	53.00	4.84
**U-87**	**20**	92.49	5.55	99.97	2.02	91.47	1.55	99.38	3.26	82.61	6.94
	**60**	77.08	10.16	101.03	3.29	88.72	2.65	99.89	3.09	79.86	3.78
	**180**	53.06	4.39	95.16	2.69	86.18	2.02	99.32	1.63	70.18	5.75
	**540**	47.99	6.03	96.81	7.42	86.97	6.13	98.85	3.52	59.77	8.42
**HeLa**	**20**	84.11	4.33	95.98	2.94	95.72	3.81	101.93	0.85	84.28	5.20
	**60**	75.36	4.73	91.05	3.59	85.63	3.88	99.86	2.90	70.44	8.82
	**180**	52.00	4.79	77.94	4.91	68.55	6.14	96.37	1.97	61.00	8.50
	**540**	50.91	2.31	72.71	4.68	60.30	8.12	91.82	2.64	54.96	8.68
**CCD 841**	**20**	85.66	6.48	100.50	2.29	95.77	3.68	101.34	2.95	98.25	4.32
	**60**	62.58	12.88	98.06	3.92	92.16	2.76	98.97	3.07	88.59	6.13
	**180**	46.60	4.54	90.40	3.16	85.26	1.53	94.45	5.48	66.22	4.37
	**540**	47.50	8.69	80.90	5.14	81.34	4.76	83.91	6.35	61.01	1.22
**MRC-5**	**20**	92.36	1.25	93.64	5.58	96.99	2.88	93.50	4.33	94.79	7.38
	**60**	78.79	5.45	92.72	4.79	91.75	3.82	88.01	2.78	86.86	6.28
	**180**	60.15	4.56	89.19	4.81	83.34	3.16	87.30	1.93	82.82	5.68
	**540**	56.13	5.27	89.19	7.67	77.21	4.81	85.23	3.21	71.26	4.66

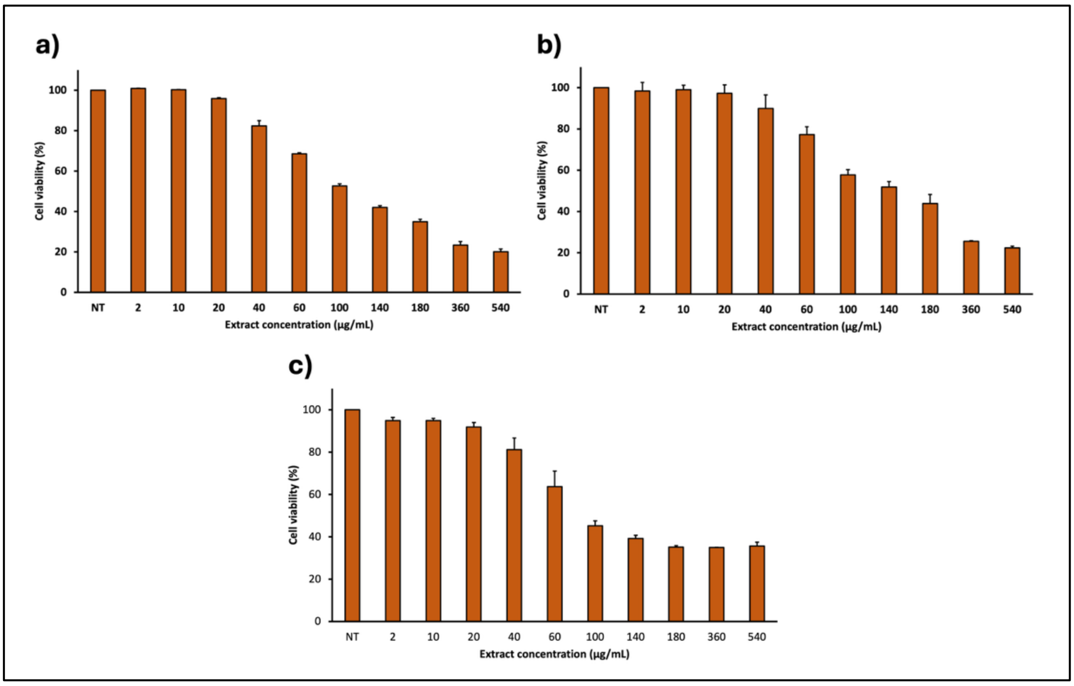

Figure S6. Cell viability of SW480 (a), E705 (b), and MDA-MB-231 (c) human cancer cell lines treated with root extract (2–540 μg/mL) for 48 h. Average values ± SEM are shown.

Table S2. Probable cytotoxicity-related biological activities of 6 compounds tentatively identified in the root extract of *A. marina* by PASS (Prediction of Activity Spectra for Substances). Pa = probable biological activity of compound; only activities with Pa > 0.7 are shown.

**Compound**	**Class**	**Cytotoxicity-related activities (Pa)**
Quercetin 3-*O*-hexoside	Flavonoid glycosides	Lipid peroxidase inhibitor (0.976)<br>TP53 expression enhancer (0.959)<br>Antineoplastic (0.833)<br>Cytostatic (0.825)<br>Caspase 3 stimulant (0.801)<br>Apotosis agonist (0.792)
Suspensaside A	Phenylethanoid glycosides	Antineoplastic (0.863)<br>Caspase 8 stimulant (0.743)
Kaempferol 3-*O*-glucoside	Flavonoid glycosides	Lipid peroxidase inhibitor (0.960)<br>TP53 expression enhancer (0.952)<br>Antineoplastic (0.834)<br>Cytostatic (0.811)<br>Caspase 3 stimulant (0.772)<br>Apoptosis agonist (0.772)
Medicoside G	Triterpene saponins	Caspase 3 stimulant (0.994)<br>Caspase 8 stimulant (0.984)<br>Trascription factor NF-kB stimulant (0.965)<br>Lipid peroxidase inhibitor (0.927)<br>ICAM1 expression inhibitor (0.908)<br>Apoptosis agonist (0.901)<br>Antineoplastic (0.870)
Esculentoside C	Triterpene saponins	Caspase 3 stimulant (0.989)

		Caspase 8 stimulant (0.986)<br>ICAM1 expression inhibitor (0.961)<br>Lipid peroxidase inhibitor (0.952)<br>Trascription factor NF-kB stimulant (0.917)<br>Antineoplastic (0.905)<br>Apoptosis agonist (0.862)<br>Antineoplastic (lung cancer) (0.807)
Azukisaponin III	Triterpene saponins	Lipid peroxidase inhibitor (0.991)<br>ICAM1 expression inhibitor (0.987)<br>Caspase 3 stimulant (0.964)<br>Caspase 8 stimulant (0.934)<br>Antineoplastic (0.908)<br>Trascription factor NF-kB stimulant (0.904)<br>Apoptosis agonist (0.883)<br>Antineoplastic (lung cancer) (0.791)
